# RNA sequencing and functional analysis implicate the regulatory role of long non-coding RNAs in tomato fruit ripening

**DOI:** 10.1093/jxb/erv203

**Published:** 2015-05-06

**Authors:** Benzhong Zhu, Yongfang Yang, Ran Li, Daqi Fu, Liwei Wen, Yunbo Luo, Hongliang Zhu

**Affiliations:** Department of Food Biotechnology, College of Food Science and Nutritional Engineering, China Agricultural University, Beijing 100083, China

**Keywords:** Fruit ripening, functional analysis, long non-coding RNA, RNA-seq, tomato, VIGS.

## Abstract

Recently, long non-coding RNAs (lncRNAs) have been shown to play critical regulatory roles in model plants, such as *Arabidopsis*, rice, and maize. However, the presence of lncRNAs and how they function in fleshy fruit ripening are still largely unknown because fleshy fruit ripening is not present in the above model plants. Tomato is the model system for fruit ripening studies due to its dramatic ripening process. To investigate further the role of lncRNAs in fruit ripening, it is necessary and urgent to discover and identify novel lncRNAs and understand the function of lncRNAs in tomato fruit ripening. Here it is reported that 3679 lncRNAs were discovered from wild-type tomato and ripening mutant fruit. The lncRNAs are transcribed from all tomato chromosomes, 85.1% of which came from intergenic regions. Tomato lncRNAs are shorter and have fewer exons than protein-coding genes, a situation reminiscent of lncRNAs from other model plants. It was also observed that 490 lncRNAs were significantly up-regulated in ripening mutant fruits, and 187 lncRNAs were down-regulated, indicating that lncRNAs could be involved in the regulation of fruit ripening. In line with this, silencing of two novel tomato intergenic lncRNAs, lncRNA1459 and lncRNA1840, resulted in an obvious delay of ripening of wild-type fruit. Overall, the results indicated that lncRNAs might be essential regulators of tomato fruit ripening, which sheds new light on the regulation of fruit ripening.

## Highlight

A relatively reliable list of tomato lncRNAs was provided. Silencing of novel lncRNAs greatly delayed the ripening of tomato fruits, implying that lncRNA might be an essential factor for fruit ripening.

## Introduction

Although genome-wide transcriptome sequencing has revealed that ~90% of eukaryotic genomes are transcribed (Wilhelm *et al.*, 2008), only 1–2% of the genome encodes proteins (Birney *et al.*, 2007), suggesting the presence of a large proportion of non-coding RNAs (ncRNAs). The ncRNAs are classified as housekeeping ncRNAs and regulatory ncRNAs (Kim and Sung, 2012). The ‘housekeeping’ ncRNAs include rRNAs, tRNAs, small nuclear RNAs (snRNAs), and small nucleolar RNAs (snoRNAs), whereas the ‘regulatory’ ncRNAs refer to small ncRNAs and long non-coding RNA (lncRNAs) (Kim and Sung, 2012; Zhu and Wang, 2012). Small ncRNAs, such as microRNAs (miRNAs) and small interfering RNAs (siRNAs), have been well studied in the last decade, as they are essential for post-transcriptional and transcriptional regulation in eukaryotes (Bonnet *et al.*, 2006; Cuperus *et al.*, 2011; Simon and Meyers, 2011). In contrast, lncRNAs are less characterized so far. It is generally believed that lncRNAs are >200 nucleotides in length and mainly transcribed by RNA polymerase II (Pol II), which are always capped, polyadenylated and frequently spliced (Ulitsky and Bartel, 2013). A few lncRNAs are generated by plant-specific Pol V, capped at the 5′ end and lacking apparent poly(A) tails. These lncRNAs function as a scaffold for the RNA-directed DNA methylation (RdDM) pathway (Kim and Sung, 2012; Wierzbicki et al., 2012). In addition, Pol III is also involved in production of lncRNAs (Wu *et al.*, 2012). With the development of next-generation sequencing, thousands of lncRNAs have been identified in model plants, such as *Arabidopsis thaliana* (Ben *et al.*, 2009; Liu *et al.*, 2012; Zhu *et al.*, 2013; Wang *et al.*, 2014), *Medicago truncatula* (Wen *et al.*, 2007), *Triticum aestivum* (Xin *et al.*, 2011), *Oryza sativa* (Li *et al.*, 2007), and *Zea mays* (Boerner and McGinnis, 2012; Li *et al.*, 2014). However, the function of lncRNAs has not been fully studied to date. Recently, the regulation mechanism of vernalization in *Arabidopsis* by COOLAIR (cool-assisted intronic non-coding RNA) and COLDAIR (cold-assisted intronic non-coding RNA), two species of lncRNAs transcribed from Flowering Locus C, has been illustrated (Swiezewski *et al.*, 2009; Heo and Sung, 2011; Sun *et al.*, 2013). LncRNAs also function as endogenous target mimics (eTMs) for a few miRNAs, which provide a new mechanism for regulation of miRNA activity (Franco-Zorrilla *et al.*, 2007; Rymarquis *et al.*, 2008; Wu *et al.*, 2013). LDMAR (long-day-specific male-fertility-associated RNA) plays an essential role in photoperiod-sensitive male sterility of rice (Ding *et al.*, 2012).

As part of the human diet, tomato has been domesticated for a few centuries (Rambla *et al.*, 2014). Today, tomato is the seventh most important crop species (after maize, rice, wheat, potatoes, soybeans, and cassava) and is the second most consumed vegetables in the world (after potatoes and before onions) (Bergougnoux, 2014). Fleshy fruit ripening of tomato is a specific morphological trait that is not present in other model plants (Bergougnoux, 2014), which means that tomato has become a model organism for basic research and applied purposes, in particular as a model system for investigations into the regulation of fruit ripening (Gapper *et al.*, 2013; Bergougnoux, 2014). However, the number, expression pattern, and characteristics of lncRNAs in tomato are still largely unknown. Therefore, it is necessary and urgent to discover and identify novel lncRNAs and understand the function of lncRNAs in tomato fruit ripening.

*ripening inhibitor* (*rin*) is one of the most famous tomato mutants that completely abolishes the normal ripening process (Vrebalov *et al.*, 2002) and thus is widely used as an excellent genetic tool in the study of fruit ripening. *RIN* was clearly elucidated as a master regulator (transcription factor) of fruit ripening to control most major ripening-related processes (Martel *et al.*, 2011; Kumar *et al.*, 2012; Qin *et al.*, 2012; Fujisawa *et al.*, 2013). In this study, a comprehensive set of lncRNAs from wild-type and *rin* mutant tomato fruit was identified using paired-end strand-specific RNA sequencing (ssRNA-Seq). In total, 3679 putative lncRNAs were discovered and were found to be distributed in every tomato chromosome; 85.1% of lncRNAs were transcribed from intergenic regions. Tomato lncRNAs are shorter, and harbour fewer exons and less coding potential than the protein-coding genes. Compared with wild-type tomato, a lot of lncRNAs showed significantly differential expression in the *rin* mutant. Moreover, down-regulation of the expression of some novel intergenic lncRNAs (lncRNA1459 and lncRNA1840) in wild-type tomato fruit induced an obvious delay of fruit ripening. These results strongly suggested that lncRNAs play an important role in the regulation of tomato fruit ripening. The findings provide new insight into the study of fruit ripening.

## Materials and methods

### Plant materials and growth conditions

Wild-type AC (*Solanum lycopersicum* cv. Ailsa Craig) and *rin* (cv. Ailsa Craig, backcross parent) tomato were grown in the greenhouse under standard greenhouse conditions (26 °C under 16h lighting, followed by 8h darkness at 20 °C), with regular additions of fertilizer and supplementary lighting when required. To collect AC fruits, they were tagged at anthesis, and harvested at the immature green (IM), mature green (MG), breaker (BR), pink (PK), and red-ripe (RR) stages, which occurred at means of 37, 42, 46, 51, and 56 days post-anthesis (DPA), respectively. Fruits of the *rin* mutant were picked at the BR stage. Immediately upon harvesting, the pericarp was manually dissected, frozen in liquid nitrogen, and stored at –80 °C. Wild-type MicroTom (*S. lycopersicum* cv. MicroTom) were also planted for virus-induced gene silencing (VIGS) in tomato fruits.

### Paired-end strand-specific RNA sequencing

Total RNA was extracted from the fruits of AC and the *rin* mutant at the BR stage (two biological replicates per genotype combined from 10 fruits each) using DeTRNa reagent (EarthOx, CA, USA) according to the manufacturer’s protocol. The RNA concentration and purity were measured using an NAS-99 spectrophotometer (ATCGene, NJ, USA). The RNA integrity was checked by agarose gel electrophoresis. Genomic DNA was removed from extracted total RNA by DNase treatment. Due to some lncRNAs lacking the poly(A) tail, total RNA was treated to remove rRNA, retaining lncRNA both with and without a poly(A) tail. The quality of the RNA and lack of contaminating rRNA were confirmed using the Agilent 2100 Bioanalyzer. Four strand-specific RNA libraries with an insert size of ~250–500 nucleotides were prepared according to a UTP method (Parkhomchuk *et al.*, 2009), and submitted to the Beijing Genomics Institute (BGI, Shenzhen, China) for 100bp paired-end sequencing on the Illumina HiSeq 2000, at a depth of ~70 million reads per library (for statistics on read counts, see Table 1). The data for this article have been deposited in the National Center for Biotechnology Information (NCBI) Sequence Read Archive (http://www.ncbi.nlm.nih.gov/sra/) under accession number SRP04432.

**Table 1.** Summary of read counts

Library	Raw reads	Clean reads	Unique clean reads
AC-1	67 698 420	66 797 294 (98.7%)	17 968 549
AC-2	69 782 776	68 165 431 (97.7%)	17 642 249
*rin*-1	70 137 108	69 247 044 (98.7%)	20 094 985
*rin*-2	72 264 600	70 614 398 (97.8%)	18 771 302

### Assembly of RNA transcripts

Sequencing reads were quality checked and trimmed to remove barcode and adaptor sequences. To rule out rRNAs, all reads were aligned to plant rRNA sequences by the Short Oligonucleotide Analysis Package (SOAP2; http://soap.genomics.org.cn/soapaligner.html). Information on plant rRNAs was extracted from the NCBI Non-Redundant (NR) data set (http://www.ncbi.nlm.nih.gov/). The clean reads from each library were aligned with the tomato reference genome (SGN release version SL2.50; ftp://ftp.sgn.cornell.edu/tomato_genome) using TopHat (version 2.0.8; http://ccb.jhu.edu/software/tophat/index.shtml). Only reads with no more than two mismatches were obtained and used to construct transcripts of each sample separately using Cufflinks (version 2.0.2; http://cole-trapnell-lab.github.io/cufflinks/) based on the tomato genome reference (SL2.50 genome).

### Bioinformatic analysis for identification of lncRNA

The assembled transcripts were compared with the tomato genome annotated protein sequences (SGN release version ITAG2.4; ftp://ftp.sgn.cornell.edu/tomato_genome) using BlastX. The non-redundant transcripts having significant alignment (*P*<1.0E-10, identity >90%, coverage >80%) with tomato proteins were excluded. For size selection, perl scripts were used to extract transcripts larger than 200 nucleotides. For the open reading frame (ORF) filter, a perl script was developed to pick up the transcripts that had short ORFs (<100 amino acids). Since a real lncRNA does not have an ORF, a putative ORF of the lncRNA candidate is defined by the longest consecutive codon chain of the lncRNA. Furthermore, to filter the transcripts containing a known protein domain, transcripts were aligned to the Protein database of KEGG (Kyoto Encyclopedia of Genes and Genomes), the NR data set, COGs (NCBI Phylogenetic classification of proteins encoded in complete genomes), and Swiss-Prot (Swiss-Protein database) using BlastX (*P*<1.0E-5, identity >90%, coverage >80%). Moreover, the resulting transcripts above were uploaded to the Coding Potential Calculator (CPC) (Kong *et al.*, 2007) to test the protein-coding potential. Only transcripts that did not pass the protein-coding score test were considered for the next step of the analysis. To rule out housekeeping ncRNAs (including tRNAs, snRNAs, and snoRNAs), all resulting transcripts were aligned to housekeeping ncRNA databases, including tRNA and snRNA sequences collected from the NCBI; and snoRNAs from the Plant snoRNAs Database (version 1.2; http://bioinf.scri.sari.ac.uk/cgi-bin/plant_snorna/home). LncRNA candidates that have significant alignment (*P*<1.0E-10, identity >90%, coverage >80%) with housekeeping lncRNAs were excluded from further analyses. To rule out miRNA precursors, putative lncRNAs were aligned against tomato miRNA precursors from miRBase (Version 21) (Kozomara and Griffiths-Jones, 2014) and from the Tomato Functional Genomics Database (TFGD). LncRNAs that have significant alignment (*P*<1.0E-10, identity >90%, coverage >80%) with miRNA precursors were excluded. Finally, the remaining transcripts were considered as tomato lncRNAs, and are listed in Supplementary Table S1 available at *JXB* online.

### Localization of lncRNAs and protein-coding genes in tomato genome

A diagram was generated to show the localization and abundance of lncRNAs and protein-coding genes in the tomato genome by the program Circos (Krzywinski *et al.*, 2009). Centromere locations were according to the report from the Tomato Genome Consortium (2012).

### Classification of lncRNAs

According to the locations relative to the nearest protein-coding genes, the annotated lncRNAs was subdivided into four categories: (i) lncRNAs without any overlap with other protein-coding genes are classified as intergenic lncRNAs (lincRNAs); (ii) lncRNAs totally in the some protein-coding loci are classified as intragenic lncRNAs; (iii) lncRNAs with some overlap with genes on the same strand, are classified as overlap lncRNAs; and (iv) antisense lncRNAs overlapping with exons of a protein-coding transcript on the opposite strand. Perl scripts were developed to classify these four categories.

### Distribution of transcript length and exon number of lncRNAs and protein-coding genes in tomato

In terms of transcript length and exon number, lncRNAs and protein-coding genes were analysed. Transcript length categories were <300, 300–400, 400–500, 500–600, 600–700, 700–800, 800–900, 900–1000, and >1000 nucleotides. Exon number categories were: 1, 2, 3, 4, 5, 6, 7, 8, 9, 10, and >10. The proportion of different kinds of lncRNAs and protein-coding transcripts was calculated.

### Prediction of miRNA targets and miRNA endogenous target mimics from lncRNAs

Tomato lncRNAs were predicted as miRNA targets using the psRNATarget (Dai and Zhao, 2011) and psRobot (H.J. Wu *et al.*, 2012). The miRNA eTMs from tomato lncRNAs were predicted according to the rules of a previous study (Wu *et al.*, 2013).

### Differential expression of lncRNAs between AC and *rin*

Differentially expressed lncRNAs between AC and *rin* were identified using the cuffdiff program (Trapnell *et al.*, 2012). The fold changes were calculated via log_2_ (FPKM *rin*/FPKM AC). LncRNAs exhibiting a |fold change| ≥1 and adjusted *P*-values <0.01 were selected as differentially expressed lncRNAs.

### RNA extraction and reverse transcription

Total RNA was isolated from fruit samples using DeTRNa reagent (EarthOx, CA, USA). The RNA concentration and purity were measured using a NAS-99 spectrophotometer (ATCGene, NJ, USA). The RNA integrity was checked by agarose gel electrophoresis. Genomic DNA was removed from extracted total RNA by DNase treatment. A 2 μg aliquot of total RNA was used for cDNA synthesis using a TransScript One-Step gDNA Removal and cDNA Synthesis SuperMix kit (Trans, Beijing, China) with random primer.

### Quantitative reverse transcription–PCR (qRT–PCR)

qRT–PCR was performed using SYBR Green PCR Master Mix with a real-time PCR System CFX96 (Bio-Rad, CA, USA). qRT–PCR conditions were as follows: 95 °C for 10min, followed by 40 cycles of 95 °C for 15 s and 60 °C for 30 s. Fluorescence changes of SYBR Green were monitored automatically in each cycle, and the threshold cycle (Ct) over the background was calculated for each reaction. Samples were normalized using *Actin*, and the relative expression levels were measured using the 2^−ΔΔCt^ analysis method. Three biological replicates were performed, and the reactions were performed in triplicate for each run. Student’s *t*-test was used to determine whether the qRT–PCR results were statistically different from two samples (*P<0.05; **P<0.01). Duncan’s multiple range test was used for three samples (*P*<0.01). Oligonucleotide primers used are listed in Supplementary Table S2 at *JXB* online.

### VIGS of tomato fruits

VIGS of MicroTom fruit was performed using *Tobacco rattle vius* (TRV) according to a previous study (Fu *et al.*, 2005). lncRNA fragments and *RIN* fragments of 300–500bp were anlysised using the VIGS tool (http://solgenomics.net/tools/vigs) to avoid off-target silencing and then amplified from tomato cDNA with PCR. A pTRV2-lncRNA or *RIN* construct was generated by inserting the *Eco*RI-digested PCR fragment of lncRNA or *RIN* into the pTRV2 vector. *Agrobacterium* strain GV3101 containing pTRV1, pTRV2, and pTRV2-lncRNA vectors were grown at 28 °C in LB medium (pH 5.6) containing 10mM MES and 20 μM acetosyringone with kanamycin, gentamycin, and rifampicin antibiotics. After shaking for 12h, cultures were harvested and resuspended in infiltration buffer (10mM MgCl_2_, 200 μM acetosyringone, 5% sucrose) to a final OD_600_ of 1.0. Resuspensions of pTRV1 and pTRV2 or pTRV2-lncRNA were mixed at a ratio of 1:1 and left at room temperature for 3h. *Agrobacterium* was infiltrated into the carpopodium of fruits with a 1ml syringe. Tomato fruits infiltrated with pTRV1 and pTRV2 were used as controls. Each inoculation was carried out three times,and each time six different plants were infiltrated. When the VIGS phenotype was visible, different sections of tomato fruits were collected and stored at –80 °C.

### RNA isolation, digestion, and RT–PCR

Poly(A)-enriched [poly(A)^+^] and poly(A)-depleted [poly(A)^–^] RNAs were isolated from total RNAs of tomato fruit using an Oligotex mRNA Mini Kit (Qiagen, CA, USA). For RNA digestion, total RNAs from tomato fruits were divided into each of four tubes and were treated as follows. First, the RNAs were incubated for 1h at 37 °C with or without enzymes: tube 1 and tube 2 with buffer only; tube 3 with T4 polynucleotide kinase (New England Biolabs, MA, USA); and tube 4 with 5′ pyrophosphohydrolase (New England Biolabs. After ethanol precipitation, RNAs in tube 1 were incubated for 1h at 37 °C with buffer only, whereas RNAs in the other three tubes were incubated with 5′ to 3′ exoribonuclease, XRN-1 (New England Biolabs), for 1h at 37 °C. The RNAs were extracted before being subjected to RT–PCR.

cDNA synthesis was performed on total RNAs, poly(A)^+^, poly(A)^–^, and different RNA digestion fractions using a TransScript One-Step gDNA Removal and cDNA Synthesis SuperMix kit (Trans, Beijing, China) with random primers. PCR was performed using EasyTaq PCR SuperMix (Trans) with PCR system T-100 (Bio-Rad). PCR conditions for *RIN*, lncRNA1459, or lncRNA1840 were as follows: 94 °C for 3min, followed by 30 cycles of 94 °C for 20 s, 55 °C for 30 s, and 72 °C for 20 s. All PCR data presented are representative of three independent experiments. Oligonucleotide primers used are listed in Supplementary Table S1 at *JXB* online. *RIN* served as a positive control for poly(A)^+^ RNAs. PCR with genomic DNA was used as a positive control. Reverse transcription which is performed in the absence of reverse transcriptase served as a negative control.

## Results

### Genome-wide discovery of lncRNAs in tomato fruit

**Fig. 1.**
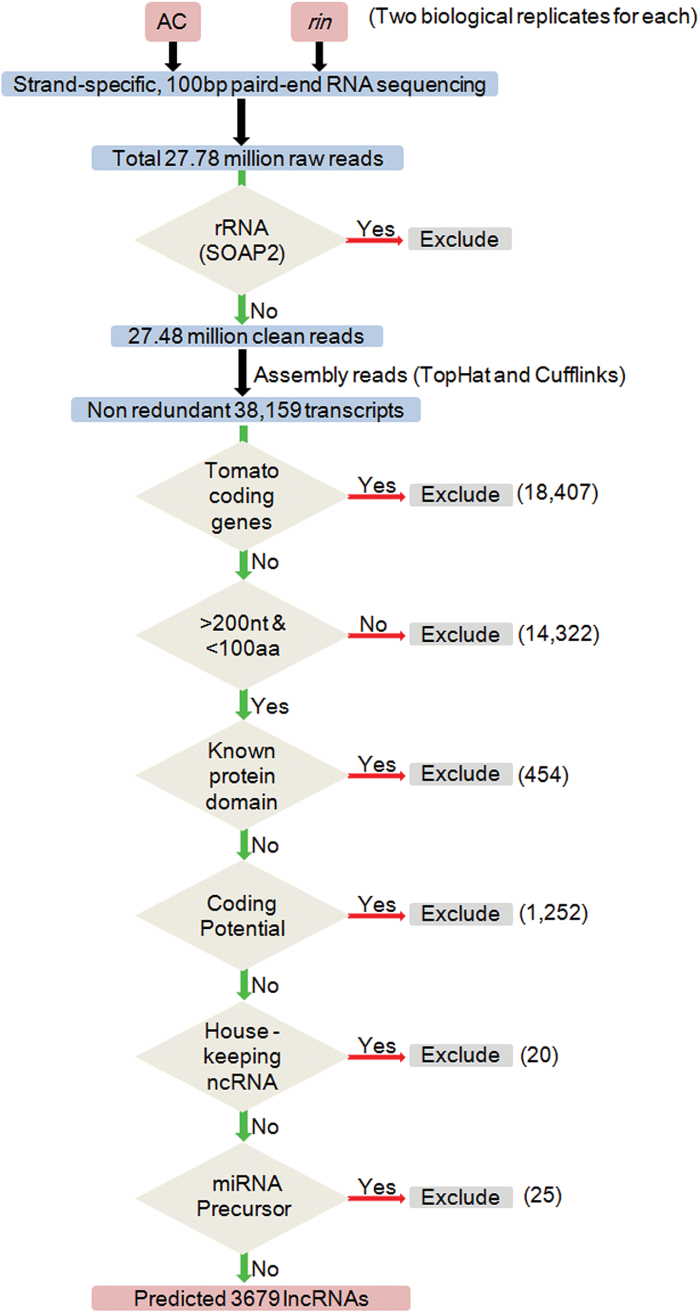
Detailed schematic diagram of the informatics pipeline for identification of tomato lncRNAs. Paired-end strand-specific RNA-Seq was performed for AC and *rin* fruits at the BR stage. Clean reads were mapped and assembled according to the known tomato genome using TopHat and Cufflinks. Transcripts were filtered with the six criteria for identification of putative lncRNAs. (i) Not tomato coding genes; (2) length >200 nucleotides and ORF <100 amino acids; (iii) not encoding known protein domains; (iv) little coding potential; (v) not housekeeping ncRNAs; and (vi) not miRNA precursors. At each step, a green arrow indicates those transcripts which were passed by the filter; a red arrow, those that were excluded. The number of transcripts that di not pass the filter is shown.

Paired-end ssRNA-Seq has become a powerful tool for the discovery of lncRNA (Ilott and Ponting, 2013), but also facilitates identification of transcript orientation. To identify lncRNAs in tomato fruits, paired-end ssRNA-Seq of transcripts from AC and *rin* fruits at the BR stage was performed in two biological replicates. A total of ~28 million clean reads was obtained (Table 1; Fig.1). A total of 38 159 unique transcripts were assembled from high-throughput RNA-Seq data from AC and *rin* fruits (Fig. 1).

To distinguish lncRNA candidates, six sequential stringent filters to the 38,159 transcripts were employed (Fig. 1). First, these transcripts were filtered with tomato coding gene sequences. Almost 48% (18,407) of transcripts were coding genes, and the remaining 52% (19,752) of transcripts might be non-coding, which was consistent with other studies showing that ncRNAs were widely transcribed (Heo *et al.*, 2013). It is generally believed that lncRNAs are longer than 200 nucleotides in size and might have a short ORF but not be able to encode polypeptides longer than 100 amino acids (Boerner and McGinnis, 2012; Liu *et al.*, 2012; Li *et al.*, 2014; Shuai *et al.*, 2014). This filter was then applied to the 19,752 transcripts, and 5430 transcripts were recovered (Fig. 1). The transcripts that might encode conserved protein domains were further filtered by comparing them with the four protein databases (KEGG, NR, COGs, and Swiss-Prot), and 4976 transcripts were obtained (Fig. 1). Next, the CPC was used to assess the protein-coding potential in order to eliminate 1252 possible coding transcripts (Fig. 1). After employing four stringent criteria, 3724 transcripts were considered as putative lncRNAs. Because housekeeping ncRNA (tRNAs, snRNAs, and snoRNAs) and miRNA precursors are two specific species of lncRNAs that function differently from other lncRNAs, the putative lncRNAs were next aligned to comprehensive sets of housekeeping ncRNAs and miRNA precursors sequences (for details, see the Materials and methods) to filter out 20 and 25 transcripts, respectively (Fig. 1). Thus, a total set of 3679 transcripts (3981 isoforms) were obtained and defined as tomato lncRNAs (Fig. 1).

### LncRNAs were widely transcribed from every tomato chromosome

**Fig. 2.**
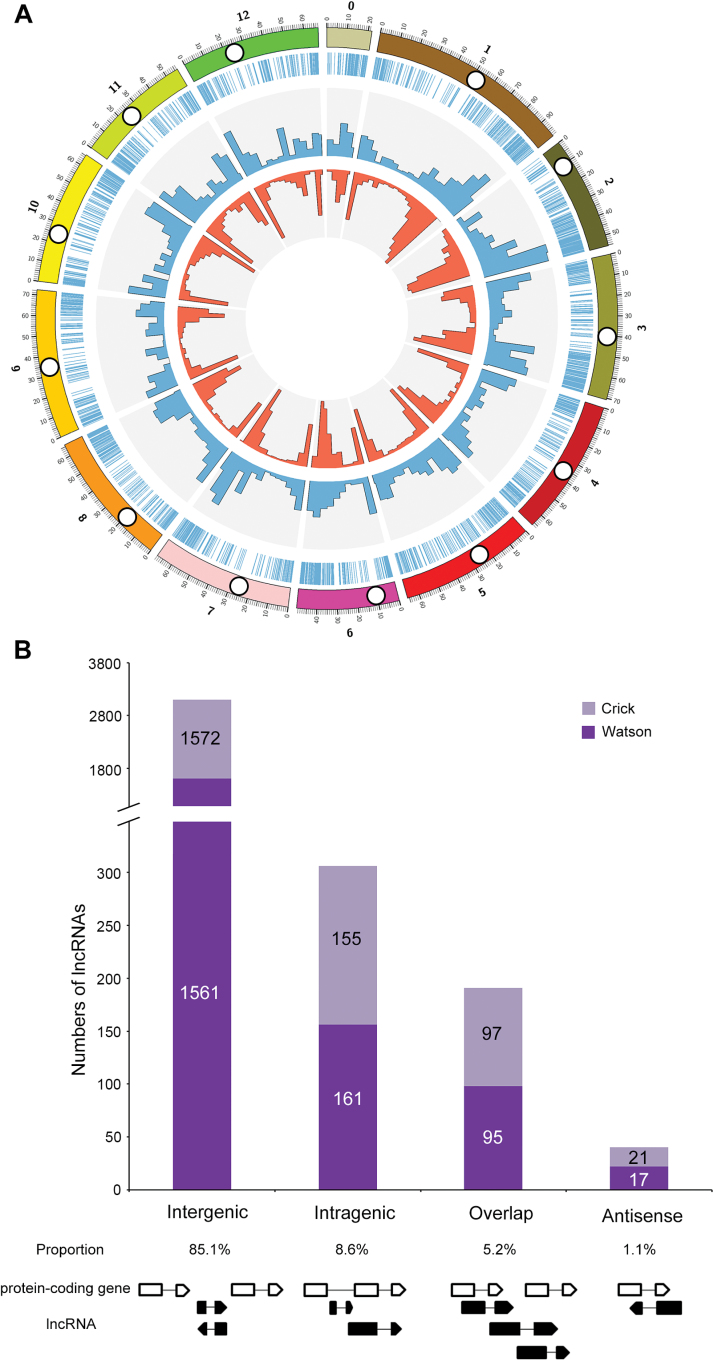
Distribution and classification of 3679 tomato lncRNAs. (A) Genome-wide distribution of tomato lncRNAs compared with that of protein-coding genes. Chromosomes 0–12 (SL2.50 genome) are shown with different colours and in a circular form as the outer thick track. The inner chromosome scale (Mb) is labelled on each chromosome. White circles show approximate centromere locations. On the second track (outer to inner), each vertical blue line reports the location of lncRNAs throughout the whole tomato genome. For the next two tracks, the abundance of lncRNAs and protein-coding genes in physical bins of 10Mb for each chromosome are shown by blue and red columns, respectively. (B) Classification of tomato lncRNAs according to its genomic position and overlap with protein-coding genes. Numbers of lncRNAs in the Watson or Crick strand for each of the four main classes were labelled on the columns (intergenic, intragenic, overlap, and antisense lncRNAs). The proportion of the four kinds of lncRNAs was calculated. A scheme of the position of the lncRNA (black box) relative to neighbouring genes (black empty box) is shown at the bottom.

Next the lncRNAs were mapped onto the recently released tomato reference genome (Tomato Genome Consortium, 2012). A Circos plot clearly showed that tomato lncRNAs were not evenly distributed across chromosomes (Fig. 2A). Similar to protein-coding genes, lncRNAs have lower densities in the pericentromeric heterochromatin regions than in the euchromatin (Fig. 2A). This result suggested that lncRNAs may share similar features of transcription with the protein-coding genes. In addition, some lncRNAs were transcribed from loci much closer to the telomeres than protein-coding genes. For instance, some lncRNAs were generated from the ends of chromosomes #1 and 3 (Fig. 2A).

According to the locations relative to the nearest protein-coding genes, lncRNAs were further classified into four types: intergenic, intragenic, overlap, and antisense lncRNAs (Fig. 2B). Whereas 5.2% and 8.6% of the lncRNAs either overlapped with genes or were transcribed from inside genes (most from introns), the majority of lncRNAs (85.1%) were located in intergenic regions (Fig. 2B). This observation is consistent with previous studies (Li *et al.*, 2014), further indicating that the type of lncRNA was termed as long intergenic non-coding RNA. In addition, only a small portion (1.1%) of lncRNAs are antisense of protein-coding genes. This result is unexpected as a previous study suggested that there were many antisense lncRNAs in *Arabidopsis* (Wang *et al.*, 2014). Interestingly, the numbers of the four types of lncRNAs from Watson and Crick strands were similar (Fig. 2B).

### Tomato lncRNAs are shorter and contain fewer exons than the protein-coding genes

**Fig. 3.**
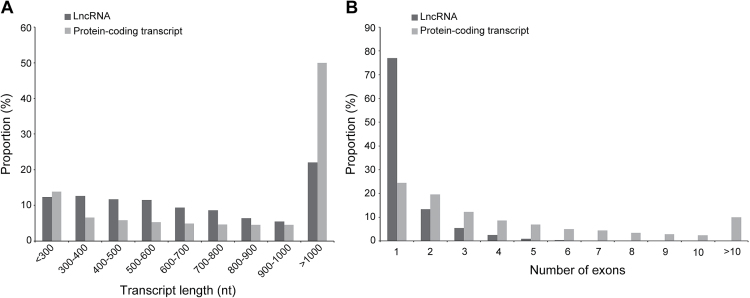
LncRNAs are shorter and have fewer exons than protein-coding transcripts. The distribution of length (A) and numbers of exons (B) of 3679 lncRNAs in comparison with 34 726 protein-coding transcripts of tomato (SL2.50 genome).

Previous studies have shown that both plant and animal lncRNAs are shorter and harbour fewer exons than protein-coding genes (Pauli *et al.*, 2012; Li *et al.*, 2014; Shuai *et al.*, 2014). To determine whether tomato lncRNAs share these features, the distribution of length and exon number of 3679 lncRNAs were analysed compared with all tomato predicted protein-coding transcripts (34,726 genes from the SL2.50 genome). Figure 3A shows that ~78% of lncRNAs ranged in size from 200 to 1000 nucleotides, with only 22% >1000 nucleotides. In contrast, for the protein-coding transcripts, ~50% of them were >1000 nucleotides. Interestingly, most (90%) of the genes encoding tomato lncRNAs only contained one or two exons, while the number of exons for the protein-coding genes ranged from one to ≥10 (Fig. 3B). These results together indicated that, unlike protein-coding genes, the majority of the tomato lncRNAs are relatively shorter and contain fewer exons.

### Predicted interactions between miRNAs and lncRNAs

**Fig. 4.**
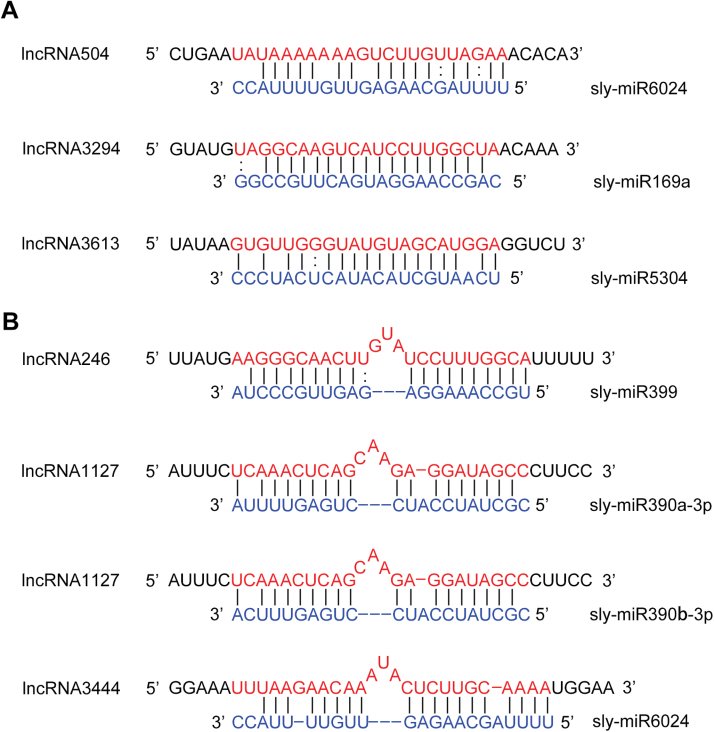
Predicted miRNA targets and endogenous target mimcs from lncRNAs. (A) Three lncRNAs as miRNA targets. (B) Three lncRNAs as endogenous target mimics of miRNAs. The blue sequences are tomato miRNAs. The red sequences indicate the regions which were complemented with miRNAs.

Interplay between miRNAs and lncRNAs is one of the important functional patterns seen for lncRNAs (Yoon *et al.*, 2014). LncRNAs could be targeted by miRNAs (Shuai *et al.*, 2014) and could also function as eTMs of miRNAs (Wu *et al.*, 2013). To examine whether lncRNAs are bona fide targets for miRNAs, all of the 3679 lncRNAs we checked using psRNATarget and psRobot. Only three miRNA targets were identified (Fig. 4A). The recovery of a small number of miRNA targets probably resulted from the low expression level of lncRNAs that were not detected by RNA-Seq. Of these three miRNA targets, lncRNA504 was the target of sly-miR6024 that was involved in plant immunity (F. Li *et al.*, 2012). LncRNA3613 was the target of sly-miR5304 that has been only identified in solanaceous plants (Gu *et al.*, 2014). In addition, lncRNA3294 was the target of sly-miR169 that is engaged in drought tolerance of tomato (Zhang *et al.*, 2011).

Although eTMs are suggested to be widespread in *Arabidopsis* (Wu *et al.*, 2013), here only three eTMs from lncRNAs were predicted (Fig. 4B). LncRNA246 was the eTM of sly-miR399, a miRNA that plays an important role in regulating phosphate homeostasis (Kuo and Chiou, 2011. LncRNA1127 is the eTM of sly-miR390-3p, which accumulates more than sly-miR390-5p (Kravchik *et al.*, 2014). Interestingly, in the case of sly-miR6024, it not only targeted lncRNA504 but also was targeted by the eTM of lncRNA3444 (Fig. 4).

### Identification of ripening-related lncRNAs

**Fig. 5.**
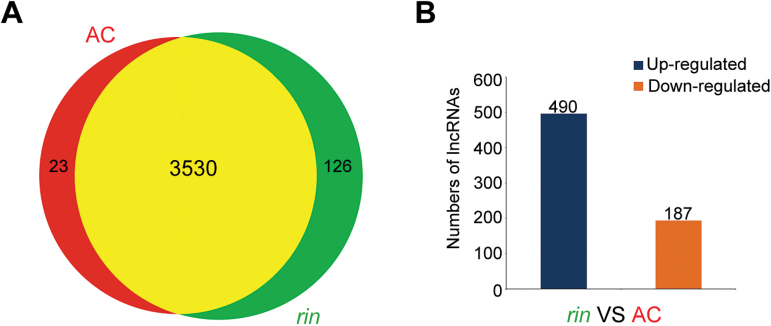
Differential expression of lncRNAs between AC and *rin* fruits. (A) Venn diagram showing non-overlap and overlap between putative lncRNAs from AC (red) and *rin* (green). The numbers of lncRNAs in different parts are shown. (B) Compared with those in AC fruits, 490 lncRNAs were up-regulated and 187 lncRNAs were down-regulated in *rin*.

**Fig. 6.**
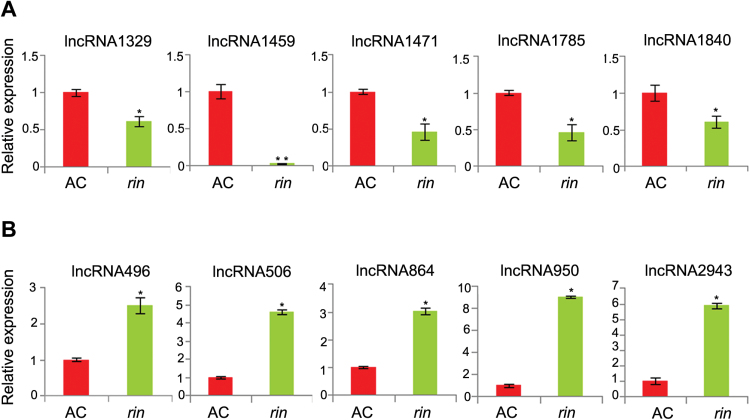
qRT–qPCR validation of RNA-Seq data on accumulation of 10 arbitrarily selected ripening-related lncRNAs. Down-regulated lncRNAs (A) and up-regulated lncRNAs (B) in *rin* according to RNA-Seq data were quantified. *Actin* expression values were used as the internal reference. The relative level of lncRNA transcripts was normalized to that in AC fruits where the amount was arbitrarily assigned a value of 1. Error bars indicate ±SD of three biological replicates, each measured in triplicate. Asterisks indicate a significant difference as determined by Student’s *t*-test (*P<0.05; **P<0.01).

Because the *rin* mutant showed a strong non-ripening phenotype compared with AC, it was hypothesized that there might be some novel ripening-related lncRNAs present in *rin*. Bioinformatics analysis revealed that 3530 of the 3679 tomato lncRNAs were accumulated in both AC and *rin* (Fig. 5A). Only 23 and 126 lncRNAs were expressed specifically in AC or *rin*, respectively (Fig. 5A). To identify further ripening-related lncRNAs, the levels of lncRNAs were compared between AC and *rin*. A total of 677 lncRNAs were significantly differentially expressed between AC and *rin*. Compared with AC, 490 of 677 lncRNAs were up-regulated in *rin*, and the other 187 lncRNAs were down-regulated (Fig. 5B). To investigate whether these differentially expressed lncRNAs are engaged in fruit ripening, 10 of them were arbitrarily selected, five from a highly up-regulated group and five from a down-regulated group. The differences in their expression levels observed by RNA-Seq were experimentally validated by qRT–PCR (Fig. 6). In addition, the fold change in the lncRNA expression level of qRT–PCR and RNA-Seq was closely correlated (*R* ^2^=0.76, *P*<0.001) (Supplementary Fig. S1 at *JXB* online). These results indicated that these lncRNAs were indeed ripening-related lncRNAs in tomato fruits, further suggesting that these lncRNAs are likely to play some roles in fruit ripening.

### Silencing of novel ripening-related intergenic lncRNAs greatly delayed the ripening of fruits

**Fig. 7.**
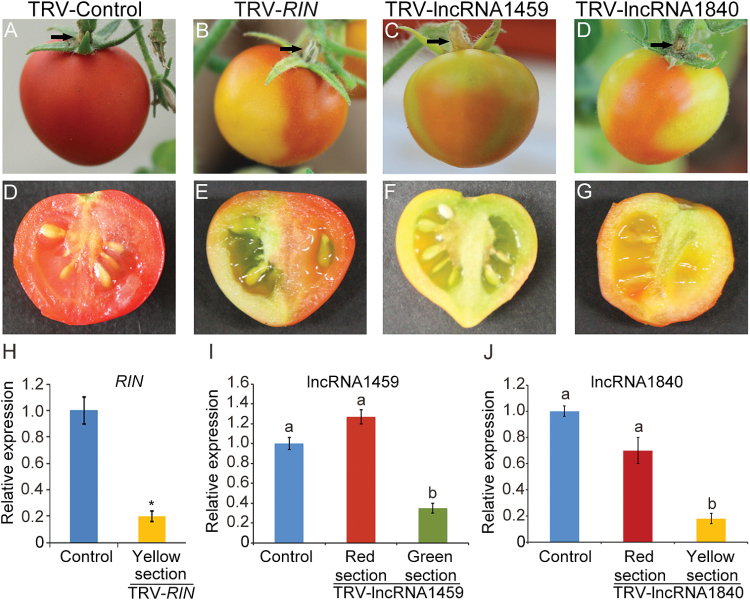
Silencing of novel intergenic lncRNAs delayed the ripening of fruits. After 2 or 3 weeks, tomato fruits infiltrated with TRV-*RIN* (B), TRV-lncRNA1459 (C), and TRV-lncRNA1840 (D) showed partial ripening compared with TRV control tomato fruit (A). Black arrows indicated the sites of injection on the carpopodium of tomato fruits. (D–G) Close up of TRV control, *RIN* silenced, lncRNA1459 silenced, and lncRNA1840 silenced fruits, respectively. (H) qRT–PCR analysis of *RIN* transcript in TRV control and TRV-*RIN* tomato fruits (yellow sections). (I) qRT–PCR analysis of lncRNA1459 transcript in TRV control and TRV-lncRNA1459 tomato fruits (red and green sections). (J) qRT–PCR analysis of lncRNA1840 transcript in TRV control and TRV-lncRNA1840 tomato fruits (red and yellow sections). *Actin* expression values were used for internal reference. The relative level of lncRNA transcripts was normalized to that in TRV control plants where the amount was arbitrarily assigned a value of 1. Error bars indicate ±SD of three biological replicates, each measured in triplicate. Data in columns with different letters are statistically different according to Duncan’s multiple range test at *P*<0.01. Asterisks indicate a significant difference as determined by Student’s *t*-test (*P<0.01).

Compared with AC fruits, accumulation of intergenic lncRNA1459 and lncRNA1840 was lower in the *rin* mutant than in AC (Fig. 6A). It was hypothesized that lncRNA1459 and lncRNA1840 may regulate the ripening process of tomato fruits. To test this hypothesis, VIGS was performed to silence lncRNA1459 and lncRNA1840 in Micro-Tom fruits. VIGS of *RIN* was used as a positive control. Intriguingly, 2 or 3 weeks after infiltration, compared with TRV control fruits (already ripening) (Fig. 7A, D), tomato fruits injected with TRV-*RIN*, TRV-lncRNA1459, or TRV-lncRNA1840 showed partial ripening, with a ripening section (red) and a non-ripening section (green or yellow) (Fig. 7B–D, E–G). Semi-quantitative PCR analysis suggested that a recombinant virus could spread from carpopodiums to fruits (Supplementary Fig. S2 at *JXB* online), and then induce the VIGS of lncRNAs or *RIN* in tomato fruits. For positive VIGS control, the expression of *RIN* was decreased to 20% in the yellow section of tomato fruits (Fig. 7H). Compared with TRV control fruits, the transcript level of lncRNA1459 in the green sections of TRV-lncRNA1459 fruits was dramatically decreased by 65% (Fig. 7I). On the other hand, the expression of lncRNA1459 in the red sections of TRV-lncRNA1459 fruits was comparable with that in TRV control fruits. Similarly, the level of lncRNA1840 in yellow sections of TRV-lncRNA1840 fruits decreased to 18% compared with TRV control fruits (Fig. 7J). Therefore, the non-ripening phenotype of TRV-lncRNA1459 or TRV-lncRNA1840 fruits clearly resulted from the silencing of lncRNA1459 or lncRNA1840. The result strongly suggested that the two novel lncRNAs were involved in the regulation of tomato fruit ripening and might play an essential role in fruit ripening.

### Features of lncRNA1840 and lncRNA1459 transcripts

**Fig. 8.**
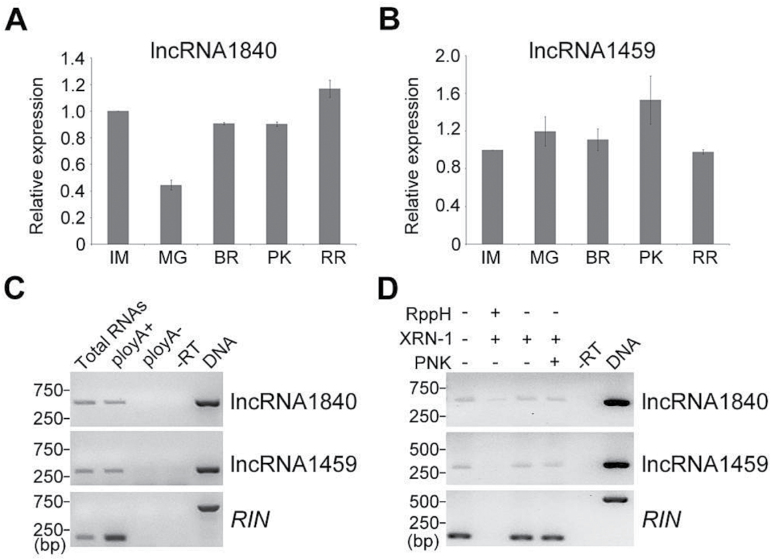
Features of lncRNA1840 and 1459. Analysis of expression of lncRNA1840 (A) and lncRNA1459 (B) during fruit ripening. *Actin* expression values were used for internal reference. The relative level of lncRNA transcripts was normalized to that at the IM stage of AC fruits where the amount was arbitrarily assigned a value of 1. Error bars indicate ±SD of three biological replicates, each measured in triplicate. IM, immature green; MG, mature green; BR, breaker; PK, pink; RR, red-ripe stage. (C) Determination of the 3′ end structure of lncRNAs. Random-primed RT–PCR was performed on total RNAs, poly(A)^+^, and poly(A)^–^ RNAs from tomato fruits to detect novel lncRNAs. (D) Analysis of the 5′ end structure of lncRNAs. Total RNAs from tomato fruits were treated (+) or not (–) with various enzymes and subjected to random-primed RT–PCR to detect specific lncRNAs. RppH, 5′ pyrophosphohydrolase; XRN-1, 5′ to 3′ exoribonuclease; PNK, polynucleotide kinase. –RT, reverse transcription was performed in the absence of reverse transcriptase. Transcript from *RIN* was detected by RT–PCR as a control for poly(A)^+^ RNA and capped RNA. PCRs with genomic DNA were used as positive controls. The amplification region of *RIN* primers contained one intron which results in a larger PCR product of DNA template than the others.

For further characterization of lncRNA1840 and lncRNA1459, the expression pattern of thse lncRNAs during fruit ripening was first explored. The transcripts of lncRNA1840 accumulated to a high level at the IM stage, and then the expression levels dropped to the lowest at the MG stage. As the fruit became red, lncRNA1840 increased rapidly up to the RR stage (Fig. 8A). However, expression of lncRNA1459 increased during fruit ripening, peaking at the PK stage (Fig. 8B). The transcriptional patterns of both lncRNA1840 and lncRNA1459 indicated that they were ripening related.

Most lncRNAs are transcribed by Pol II, and polyadenylation is part of the maturation process of Pol II-dependent transcripts. In order to determine whether lncRNA1840 or lncRNA1459 has a poly(A) tail, total RNAs of tomato fruits were separated into poly(A)^+^ and poly(A)^–^ fractions followed by the detection of lncRNAs by RT–PCR. As shown in Fig. 8C, *RIN* was detected only from total and poly(A)^+^ RNA, which is a positive control of poly(A)^+^ RNA. lncRNA1840 and lncRNA1459 were also detectable from total and poly(A)^+^ RNAs, but not from poly(A)^–^ RNAs, suggesting these that two lncRNAs had poly(A) tails and might be Pol II-dependent transcripts.

Normally, 5′ initiating nucleotides of Pol II transcripts have 7-methylguanosine caps. To determine the 5′ end structure of lncRNA1840 and lncRNA1459, various enzymatic treatments of total RNAs were performed. Total RNAs were treated with 5′ pyrophosphohydrolase (RppH), which decaps RNA and converts the 5′ 7-methylguanylate cap or 5′ triphosphate to 5′ monophosphate, or T4 polynucleotide kinase (PNK), which adds a 5′ phosphate group to 5′ hydroxyl RNAs. The RNAs were then digested with XRN-1, a 5′ to 3′ exoribonuclease that acts on RNAs only with a 5′ monophosphate group. Next, random primed RT–PCR was conducted on these treated RNA samples to detect lncRNA1840 or lncRNA1459. The RNA samples treated with PNK and XRN-1 or XRN-1 alone showed a transcript level of lncRNA1840 and lncRNA1459 comparable with RNAs with no treatment, suggesting that lncRNA1840 and lncRNA1459 did not have a 5′ monophosphate group (Fig. 8D). Furthermore, dramatic reduction in the abundance of lncRNA1840 and lncRNA1459 was only found in RNAs treated with RppH followed by XRN-1 (Fig. 8D), and lncRNA1840 and lncRNA1459 were polyadenylated (Fig. 8C), together indicating that lncRNA1840 and lncRNA1459 might have a 5′ 7-methylguanylate cap, but not a 5′ triphosphate group.

## Discussion

### A reliable list of lncRNAs from tomato fruits

Recent studies revealed that lncRNAs exert a crucial role in various biological processes of plants (Zhang and Chen, 2013). Although many lncRNAs have been identified from model plants, such as *Arabidopsis* (Ben *et al.*, 2009; Liu *et al.*, 2012; Zhu *et al.*, 2013; Wang *et al.*, 2014), wheat (Xin *et al.*, 2011), rice (Li *et al.*, 2007), and maize (Boerner and McGinnis, 2012; Li *et al.*, 2014), much work still remains to be done with tomato. In the present study, a total of 3679 lncRNA loci (3981 isoforms) were identified in tomato, a model plant for study of fruit ripening (Fig. 1). Although the strict criteria pipeline for identification of tomato lncRNAs is similar to that used in previous studies in plants (Ben *et al.*, 2009; Boerner and McGinnis, 2012; Zhu *et al.*, 2013; Li *et al.*, 2014; Shuai *et al.*, 2014), there are several advantages to the present list of lncRNAs. (i) The lncRNAs should include some tomato lncRNAs that lack polyadenylation as the RNA-Seq data were obtained from RNAs that were depleted only of rRNA, but not of non-polyadenylated RNAs. In line with this, many lncRNAs without poly(A) tails have recently been identified from poly(A)^–^ RNA-Seq in *Arabidopsis* (Di *et al.*, 2014). (ii) Another ubiquitous limitation for recent lncRNA studies is that strand information from RNA-Seq data was missing (Li *et al.*, 2012; Kumar *et al.*, 2013; Shuai *et al.*, 2014). Here, the strand-specific RNA-Seq allowed easy identification of the transcription orientation of lncRNAs, thus providing a useful resource for further functional analysis.

There are also some limitations to the list of tomato lncRNAs obtained. (i) Due to the inherent limitations of using 100bp paired-end RNA-Seq, it is difficult to obtain the complete sequences for all predicted tomato lncRNAs. (ii) Normally, 1–2% of the genome encodes proteins (Birney *et al.*, 2007); however, 48% of the assembled transcripts had significant homology with tomato protein-coding genes from the first filter of strict criteria (Fig. 1). These results indicated that the RNA-Seq was not deep enough to recover tomato lncRNAs fully. (iii) Some previously characterized lncRNAs have the potential to encode peptides longer than 100 amino acids, such as HOTAIR (HOX transcript antisense RNA), XIST (X-inactive specific transcript), and KCNQ1OT1 (KCNQ1 overlapping transcript 1) (Li *et al.*, 2014). However these type of lncRNAs could not meet the relatively strict criteria used here for definition as lncRNAs and thus were probably filtered out in the screening.

In summary, although some tomato lncRNAs might be excluded due to the sequencing limitations and strict bioinformatics criteria, a relatively robust and reliable list of tomato lncRNAs is provided. The list of lncRNAs will probably be very useful for other researchers.

### Differential expression of lncRNAs was related to fruit ripening

Transcriptomic sequencing on different varieties of tomato revealed the presence of a large number of ncRNAs (Tomato Genome Consortium, 2012; Aoki *et al.*, 2013). Previous studies have already shown that small RNAs are involved in the regulation of tomato fruit (Moxon *et al.*, 2008; Mohorianu *et al.*, 2011; Zuo *et al.*, 2012; Karlova *et al.*, 2013). Differential accumulation of small RNAs during tomato fruit ripening indicated that the regulation mechanism of small RNAz for fruit ripening would be complicated. Furthermore, degradome sequencing of tomato fruit revealed that a number of miRNA targets were genes which were previoulsy already characterized as important factors in tomato fruit ripening (Karlova *et al.*, 2013). For example, the target of miR156/157 is *Colorless Non-Ripening* (*CNR*) (Karlova *et al.*, 2013), an epigenetic mutation of which could inhibit tomato fruit ripening (Manning *et al.*, 2006). The target of miR172 is *APETALA2* (*AP2*) (Karlova *et al.*, 2013), which is a negative regulator of tomato fruit ripening (Chung *et al.*, 2010; Karlova *et al.*, 2011). In contrast, ripening-related lncRNAs have not been as comprehensively identified and functionally examined in tomato. The present RNA-Seq data and further qRT–PCR analysis revealed that many lncRNAs were significantly differentially expressed in the *rin* mutant compared with AC (Figs 5, 6). The result clearly suggested that lncRNAs might be involved in the regulation of tomato fruit ripening.

### Functional identification of ripening-related lncRNAs is critical

Because the transcription machinery is not perfect, there are large numbers of spurious RNAs that might be by-products (Struhl, 2007). Recently many studies discovered thousands of lncRNAs from many plant species; however, they did not suggest that they are functional because ‘lncRNA’ is only a name for transcripts. The current dogma is that a few lncRNAs are functional and most are not (Ulitsky and Bartel, 2013); therefore, functional identification and assignment would be important for these lncRNAs. Due to the numbers of ripening-related lncRNAs that were identified in the present study, a rapid and high-throughput method would be necessary for their functional characterization.

VIGS is a widely used tool to identify gene function in tomato fruit development and ripening (Fu *et al.*, 2005; Manning *et al.*, 2006; Orzaez *et al.*, 2006, 2009; Quadrana *et al.*, 2011; Fantini *et al.*, 2013; Lange *et al.*, 2013) because it is an easy, rapid, reliable, and transformation-free method (Senthil-Kumar and Mysore, 2011; Lange *et al.*, 2013). In addition, partial sequence information of one transcript is sufficient to silence itself by VIGS. Here it was demonstrated that VIGS is a powerful tool to study lncRNAs too. VIGS of lncRNA1459 and lncRNA1840 in tomato fruits resulted in a delay of fruit ripening (Fig. 7), taken together with the expression patterns of these two lncRNAs during fruit ripening (Fig. 8), provides strong evidence that lncRNA1459 and 1840 are functional in fruit ripening regulation.

Capping RNA is critical for RNA interactions with many nuclear and cytoplasmic proteins, and plays essential roles in RNA stability, splicing, nucleocytoplasmic transport, and translation initiation to regulate RNA accumulation (Topisirovic *et al.*, 2011). Also, polyadenylation of RNA is important for nuclear export, translation, and stability of RNAs (Elkon *et al.*, 2013). End structure analysis of lncRNA1840 and lncRNA1459 indicated that they might be Pol II-dependent transcripts with 5′ caps and 3′ poly(A) tails (Fig. 8C, D), which is very helpful for further functional characterization of these two lncRNAs in the future. Thus this study sheds new light on the regulation of fruit ripening, which might trigger more comprehensive studies on tomato lncRNAs. It would be necessary to investigate further the functional motifs and target genes of lncRNAs in tomato, which would help to elucidate fully the regulatory mechanisms of lncRNAs on fruit ripening.

## Supplementary data

Supplementary data are available at *JXB* online.

Figure S1. Expression levels as determined by RNA-Seq and qRT–PCR are highly correlated.

Figure S2. Semi-quantitative PCR detection of recombinant TRV RNA in uninjected tomato fruits with infiltrated carpopodiums.

Table S1. List of lncRNAs of tomato.

Table S2. Primers used in the study.

Supplementary Data

## References

[CIT0001] Aoki K Ogata Y Igarashi K Yano K Nagasaki H Kaminuma E Toyoda A 2013 Functional genomics of tomato in a post-genome-sequencing phase. Breeding Science 63, 14–20. 23641177 10.1270/jsbbs.63.14 PMC3621439

[CIT0002] Ben AB Wirth S Merchan F 2009 Novel long non-protein coding RNAs involved in Arabidopsis differentiation and stress responses. Genome Research 19, 57–69. 18997003 10.1101/gr.080275.108 PMC2612962

[CIT0003] Bergougnoux V 2014 The history of tomato: from domestication to biopharming. Biotechnology Advances 32, 170–189. 24211472 10.1016/j.biotechadv.2013.11.003

[CIT0004] Birney E Stamatoyannopoulos JA Dutta A 2007 Identification and analysis of functional elements in 1% of the human genome by the ENCODE pilot project. Nature 447, 799–816. 17571346 10.1038/nature05874 PMC2212820

[CIT0005] Boerner S McGinnis KM 2012 Computational identification and functional predictions of long noncoding RNA in Zea mays. PLoS One 7, e43047. 22916204 10.1371/journal.pone.0043047 PMC3420876

[CIT0006] Bonnet E Van de Peer Y Rouze P 2006 The small RNA world of plants. New Phytologist 171, 451–468. 16866953 10.1111/j.1469-8137.2006.01806.x

[CIT0007] Chung MY Vrebalov J Alba R Lee J McQuinn R Chung JD Klein P Giovannoni J 2010 A tomato (Solanum lycopersicum) APETALA2/ERF gene, SlAP2a, is a negative regulator of fruit ripening. The Plant Journal 64, 936–947. 21143675 10.1111/j.1365-313X.2010.04384.x

[CIT0008] Cuperus JT Fahlgren N Carrington JC 2011 Evolution and functional diversification of MIRNA genes. The Plant Cell 23, 431–442. 21317375 10.1105/tpc.110.082784 PMC3077775

[CIT0009] Dai X Zhao PX 2011 psRNATarget: a plant small RNA target analysis server. Nucleic Acids Research 39, W155–W159. 21622958 10.1093/nar/gkr319 PMC3125753

[CIT0010] Di C Yuan J Wu Y 2014 Characterization of stress-responsive lncRNAs in Arabidopsis thaliana by integrating expression, epigenetic and structural features. The Plant Journal 80, 848–861. 25256571 10.1111/tpj.12679

[CIT0011] Ding J Lu Q Ouyang Y Mao H Zhang P Yao J Xu C Li X Xiao J Zhang Q 2012 A long noncoding RNA regulates photoperiod-sensitive male sterility, an essential component of hybrid rice. Proceedings of the National Academy of Sciences, USA 109, 2654–2659. 10.1073/pnas.1121374109 PMC3289353 22308482

[CIT0012] Elkon R Ugalde AP Agami R 2013 Alternative cleavage and polyadenylation: extent, regulation and function. Nature Reviews Genetics 14, 496–506. 10.1038/nrg3482 23774734

[CIT0013] Fantini E Falcone G Frusciante S Giliberto L Giuliano G 2013 Dissection of tomato lycopene biosynthesis through virus-induced gene silencing. Plant Physiology 163, 986–998. 24014574 10.1104/pp.113.224733 PMC3793073

[CIT0014] Franco-Zorrilla JM Valli A Todesco M Mateos I Puga MI Rubio-Somoza I Leyva A Weigel D Garcia JA Paz-Ares J 2007 Target mimicry provides a new mechanism for regulation of microRNA activity. Nature Genetics 39, 1033–1037. 17643101 10.1038/ng2079

[CIT0015] Fu DQ Zhu BZ Zhu HL Jiang WB Luo YB 2005 Virus-induced gene silencing in tomato fruit. The Plant Journal 43, 299–308. 15998315 10.1111/j.1365-313X.2005.02441.x

[CIT0016] Fujisawa M Nakano T Shima Y Ito Y 2013 A large-scale identification of direct targets of the tomato MADS box transcription factor RIPENING INHIBITOR reveals the regulation of fruit ripening. The Plant Cell 25, 371–386. 23386264 10.1105/tpc.112.108118 PMC3608766

[CIT0017] Gapper NE McQuinn RP Giovannoni JJ 2013 Molecular and genetic regulation of fruit ripening. Plant Molecular Biology 82, 575–591. 23585213 10.1007/s11103-013-0050-3

[CIT0018] Gu M Liu W Meng Q Zhang W Chen A Sun S Xu G 2014 Identification of microRNAs in six solanaceous plants and their potential link with phosphate and mycorrhizal signalings. Journal of Integrative Plant Biology 56, 1164–1178. 24975554 10.1111/jipb.12233

[CIT0019] Heo JB Lee YS Sung S 2013 Epigenetic regulation by long noncoding RNAs in plants. Chromosome Research 21, 685–693. 24233054 10.1007/s10577-013-9392-6 PMC4049567

[CIT0020] Heo JB Sung S 2011 Vernalization-mediated epigenetic silencing by a long intronic noncoding RNA. Science 331, 76–79. 21127216 10.1126/science.1197349

[CIT0021] Ilott NE Ponting CP 2013 Predicting long non-coding RNAs using RNA sequencing. Methods 63, 50–59. 23541739 10.1016/j.ymeth.2013.03.019

[CIT0022] Karlova R Rosin FM Busscher-Lange J Parapunova V Do PT Fernie AR Fraser PD Baxter C Angenent GC de Maagd RA 2011 Transcriptome and metabolite profiling show that APETALA2a is a major regulator of tomato fruit ripening. The Plant Cell 23, 923–941. 21398570 10.1105/tpc.110.081273 PMC3082273

[CIT0023] Karlova R van Haarst JC Maliepaard C van de Geest H Bovy AG Lammers M Angenent GC de Maagd RA 2013 Identification of microRNA targets in tomato fruit development using high-throughput sequencing and degradome analysis. Journal of Experimental Botany 64, 1863–1878. 23487304 10.1093/jxb/ert049 PMC3638818

[CIT0024] Kim ED Sung S 2012 Long noncoding RNA: unveiling hidden layer of gene regulatory networks. Trends in Plant Science 17, 16–21. 22104407 10.1016/j.tplants.2011.10.008

[CIT0025] Kong L Zhang Y Ye ZQ Liu XQ Zhao SQ Wei L Gao G 2007 CPC: assess the protein-coding potential of transcripts using sequence features and support vector machine. Nucleic Acids Research 35, W345–W349. 17631615 10.1093/nar/gkm391 PMC1933232

[CIT0026] Kozomara A Griffiths-Jones S 2014 miRBase: annotating high confidence microRNAs using deep sequencing data. Nucleic Acids Research 42, D68–D73. 24275495 10.1093/nar/gkt1181 PMC3965103

[CIT0027] Kravchik M Sunkar R Damodharan S Stav R Zohar M Isaacson T Arazi T 2014 Global and local perturbation of the tomato microRNA pathway by a trans-activated DICER-LIKE 1 mutant. Journal of Experimental Botany 65, 725–739. 24376253 10.1093/jxb/ert428 PMC3904720

[CIT0028] Krzywinski M Schein J Birol I Connors J Gascoyne R Horsman D Jones SJ Marra MA 2009 Circos: an information aesthetic for comparative genomics. Genome Research 19, 1639–1645. 19541911 10.1101/gr.092759.109 PMC2752132

[CIT0029] Kumar R Sharma MK Kapoor S Tyagi AK Sharma AK 2012 Transcriptome analysis of rin mutant fruit and in silico analysis of promoters of differentially regulated genes provides insight into LeMADS-RIN-regulated ethylene-dependent as well as ethylene-independent aspects of ripening in tomato. Molecular Genetics and Genomics 287, 189–203. 22212279 10.1007/s00438-011-0671-7

[CIT0030] Kumar V Westra HJ Karjalainen J 2013 Human disease-associated genetic variation impacts large intergenic non-coding RNA expression. PLoS Genetics 9, e1003201. 23341781 10.1371/journal.pgen.1003201 PMC3547830

[CIT0031] Kuo HF Chiou TJ 2011 The role of microRNAs in phosphorus deficiency signaling. Plant Physiology 156, 1016–1024. 21562333 10.1104/pp.111.175265 PMC3135939

[CIT0032] Lange M Yellina AL Orashakova S Becker A 2013 Virus-induced gene silencing (VIGS) in plants: an overview of target species and the virus-derived vector systems. Methods in Molecular Biology 975, 1–14. 23386291 10.1007/978-1-62703-278-0_1

[CIT0033] Li F Pignatta D Bendix C Brunkard JO Cohn MM Tung J Sun H Kumar P Baker B 2012 MicroRNA regulation of plant innate immune receptors. Proceedings of the National Academy of Sciences, USA 109, 1790–1795. 10.1073/pnas.1118282109 PMC3277104 22307647

[CIT0034] Li L Eichten SR Shimizu R 2014 Genome-wide discovery and characterization of maize long non-coding RNAs. Genome Biology 15, R40. 24576388 10.1186/gb-2014-15-2-r40 PMC4053991

[CIT0035] Li L Wang X Sasidharan R 2007 Global identification and characterization of transcriptionally active regions in the rice genome. PLoS One 2, e294. 17372628 10.1371/journal.pone.0000294 PMC1808428

[CIT0036] Li T Wang S Wu R Zhou X Zhu D Zhang Y 2012 Identification of long non-protein coding RNAs in chicken skeletal muscle using next generation sequencing. Genomics 99, 292–298. 22374175 10.1016/j.ygeno.2012.02.003

[CIT0037] Liu J Jung C Xu J Wang H Deng S Bernad L Arenas-Huertero C Chua NH 2012 Genome-wide analysis uncovers regulation of long intergenic noncoding RNAs in Arabidopsis. The Plant Cell 24, 4333–4345. 23136377 10.1105/tpc.112.102855 PMC3531837

[CIT0038] Manning K Tor M Poole M Hong Y Thompson AJ King GJ Giovannoni JJ Seymour GB 2006 A naturally occurring epigenetic mutation in a gene encoding an SBP-box transcription factor inhibits tomato fruit ripening. Nature Genetics 38, 948–952. 16832354 10.1038/ng1841

[CIT0039] Martel C Vrebalov J Tafelmeyer P Giovannoni JJ 2011 The tomato MADS-box transcription factor RIPENING INHIBITOR interacts with promoters involved in numerous ripening processes in a COLORLESS NONRIPENING-dependent manner. Plant Physiology 157, 1568–1579. 21941001 10.1104/pp.111.181107 PMC3252172

[CIT0040] Mohorianu I Schwach F Jing R Lopez-Gomollon S Moxon S Szittya G Sorefan K Moulton V Dalmay T 2011 Profiling of short RNAs during fleshy fruit development reveals stage-specific sRNAome expression patterns. The Plant Journal 67, 232–246. 21443685 10.1111/j.1365-313X.2011.04586.x

[CIT0041] Moxon S Jing R Szittya G Schwach F Rusholme PR Moulton V Dalmay T 2008 Deep sequencing of tomato short RNAs identifies microRNAs targeting genes involved in fruit ripening. Genome Research 18, 1602–1609. 18653800 10.1101/gr.080127.108 PMC2556272

[CIT0042] Orzaez D Medina A Torre S Fernandez-Moreno JP Rambla JL Fernandez-Del-Carmen A Butelli E Martin C Granell A 2009 A visual reporter system for virus-induced gene silencing in tomato fruit based on anthocyanin accumulation. Plant Physiology 150, 1122–1134. 19429602 10.1104/pp.109.139006 PMC2705029

[CIT0043] Orzaez D Mirabel S Wieland WH Granell A 2006 Agroinjection of tomato fruits. A tool for rapid functional analysis of transgenes directly in fruit. Plant Physiology 140, 3–11. 16403736 10.1104/pp.105.068221 PMC1326026

[CIT0044] Parkhomchuk D Borodina T Amstislavskiy V Banaru M Hallen L Krobitsch S Lehrach H Soldatov A 2009 Transcriptome analysis by strand-specific sequencing of complementary DNA. Nucleic Acids Research 37, e123. 19620212 10.1093/nar/gkp596 PMC2764448

[CIT0045] Pauli A Valen E Lin MF 2012 Systematic identification of long noncoding RNAs expressed during zebrafish embryogenesis. Genome Research 22, 577–591. 22110045 10.1101/gr.133009.111 PMC3290793

[CIT0046] Qin G Wang Y Cao B Wang W Tian S 2012 Unraveling the regulatory network of the MADS box transcription factor RIN in fruit ripening. The Plant Journal 70, 243–255. 22098335 10.1111/j.1365-313X.2011.04861.x

[CIT0047] Quadrana L Rodriguez MC Lopez M 2011 Coupling virus-induced gene silencing to exogenous green fluorescence protein expression provides a highly efficient system for functional genomics in Arabidopsis and across all stages of tomato fruit development. Plant Physiology 156, 1278–1291. 21531899 10.1104/pp.111.177345 PMC3135922

[CIT0048] Rambla JL Tikunov YM Monforte AJ Bovy AG Granell A 2014 The expanded tomato fruit volatile landscape. Journal of Experimental Botany 65, 4613–4623. 24692651 10.1093/jxb/eru128

[CIT0049] Rymarquis LA Kastenmayer JP Huttenhofer AG Green PJ 2008 Diamonds in the rough: mRNA-like non-coding RNAs. Trends in Plant Science 13, 329–334. 18448381 10.1016/j.tplants.2008.02.009

[CIT0050] Senthil-Kumar M Mysore KS 2011 New dimensions for VIGS in plant functional genomics. Trends in Plant Science 16, 656–665. 21937256 10.1016/j.tplants.2011.08.006

[CIT0051] Shuai P Liang D Tang S Zhang Z Ye CY Su Y Xia X Yin W 2014 Genome-wide identification and functional prediction of novel and drought-responsive lincRNAs in Populus trichocarpa. Journal of Experimental Botany 65, 4975–4983. 24948679 10.1093/jxb/eru256 PMC4144774

[CIT0052] Simon SA Meyers BC 2011 Small RNA-mediated epigenetic modifications in plants. Current Opinion in Plant Biology 14, 148–155. 21159545 10.1016/j.pbi.2010.11.007

[CIT0053] Struhl K 2007 Transcriptional noise and the fidelity of initiation by RNA polymerase II. Nature Structural and Molecular Biology 14, 103–105. 10.1038/nsmb0207-103 17277804

[CIT0054] Sun Q Csorba T Skourti-Stathaki K Proudfoot NJ Dean C 2013 R-loop stabilization represses antisense transcription at the Arabidopsis FLC locus. Science 340, 619–621. 23641115 10.1126/science.1234848 PMC5144995

[CIT0055] Swiezewski S Liu F Magusin A Dean C 2009 Cold-induced silencing by long antisense transcripts of an Arabidopsis Polycomb target. Nature 462, 799–802. 20010688 10.1038/nature08618

[CIT0056] **Tomato Genome Consortium**. 2012 The tomato genome sequence provides insights into fleshy fruit evolution. Nature 485, 635–641. 22660326 10.1038/nature11119 PMC3378239

[CIT0057] Topisirovic I Svitkin YV Sonenberg N Shatkin AJ 2011 Cap and cap-binding proteins in the control of gene expression. Wiley Interdisciplinary Reviews: RNA 2, 277–298. 21957010 10.1002/wrna.52

[CIT0058] Trapnell C Roberts A Goff L Pertea G Kim D Kelley DR Pimentel H Salzberg SL Rinn JL Pachter L 2012 Differential gene and transcript expression analysis of RNA-Seq experiments with TopHat and Cufflinks. Nature Protocols 7, 562–578. 22383036 10.1038/nprot.2012.016 PMC3334321

[CIT0059] Ulitsky I Bartel DP 2013 lincRNAs: genomics, evolution, and mechanisms. Cell 154, 26–46. 23827673 10.1016/j.cell.2013.06.020 PMC3924787

[CIT0060] Vrebalov J Ruezinsky D Padmanabhan V White R Medrano D Drake R Schuch W Giovannoni J 2002 A MADS-box gene necessary for fruit ripening at the tomato ripening-inhibitor (rin) locus. Science 296, 343–346. 11951045 10.1126/science.1068181

[CIT0061] Wang H Chung PJ Liu J Jang IC Kean MJ Xu J Chua NH 2014 Genome-wide identification of long noncoding natural antisense transcripts and their responses to light in Arabidopsis. Genome Research 24, 444–453. 24402519 10.1101/gr.165555.113 PMC3941109

[CIT0062] Wen J Parker BJ Weiller GF 2007 In silico identification and characterization of mRNA-like noncoding transcripts in Medicago truncatula. In Silico Biology 7, 485–505. 18391239

[CIT0063] Wierzbicki AT 2012 The role of long non-coding RNA in transcriptional gene silencing. Current Opinion in Plant Biology 15, 517–522. 22960034 10.1016/j.pbi.2012.08.008

[CIT0064] Wilhelm BT Marguerat S Watt S Schubert F Wood V Goodhead I Penkett CJ Rogers J Bahler J 2008 Dynamic repertoire of a eukaryotic transcriptome surveyed at single-nucleotide resolution. Nature 453, 1239–1243. 18488015 10.1038/nature07002

[CIT0065] Wu HJ Ma YK Chen T Wang M Wang XJ 2012 PsRobot: a web-based plant small RNA meta-analysis toolbox. Nucleic Acids Research 40, W22–W28. 22693224 10.1093/nar/gks554 PMC3394341

[CIT0066] Wu HJ Wang ZM Wang M Wang XJ 2013 Widespread long noncoding RNAs as endogenous target mimics for microRNAs in plants. Plant Physiology 161, 1875–1884. 23429259 10.1104/pp.113.215962 PMC3613462

[CIT0067] Wu J Okada T Fukushima T Tsudzuki T Sugiura M Yukawa Y 2012 A novel hypoxic stress-responsive long non-coding RNA transcribed by RNA polymerase III in Arabidopsis. RNA Biology 9, 302–313. 22336715 10.4161/rna.19101

[CIT0068] Xin M Wang Y Yao Y Song N Hu Z Qin D Xie C Peng H Ni Z Sun Q 2011 Identification and characterization of wheat long non-protein coding RNAs responsive to powdery mildew infection and heat stress by using microarray analysis and SBS sequencing. BMC Plant Biology 11, 61. 21473757 10.1186/1471-2229-11-61 PMC3079642

[CIT0069] Yoon JH Abdelmohsen K Gorospe M 2014 Functional interactions among microRNAs and long noncoding RNAs. Seminars in Cell and Developmental Biology 34, 9–14. 24965208 10.1016/j.semcdb.2014.05.015 PMC4163095

[CIT0070] Zhang X Zou Z Gong P Zhang J Ziaf K Li H Xiao F Ye Z 2011 Over-expression of microRNA169 confers enhanced drought tolerance to tomato. Biotechnology Letters 33, 403–409. 20960221 10.1007/s10529-010-0436-0

[CIT0071] Zhang YC Chen YQ 2013 Long noncoding RNAs: new regulators in plant development. Biochemical and Biophysical Research Communications 436, 111–114. 23726911 10.1016/j.bbrc.2013.05.086

[CIT0072] Zhu QH Stephen S Taylor J Helliwell CA Wang MB 2013 Long noncoding RNAs responsive to Fusarium oxysporum infection in Arabidopsis thaliana. New Phytologist 201, 574–584. 24117540 10.1111/nph.12537

[CIT0073] Zhu QH Wang MB 2012 Molecular functions of long non-coding RNAs in plants. Genes (Basel) 3, 176–190. 24704849 10.3390/genes3010176 PMC3899965

[CIT0074] Zuo J Zhu B Fu D Zhu Y Ma Y Chi L Ju Z Wang Y Zhai B Luo Y 2012 Sculpting the maturation, softening and ethylene pathway: the influences of microRNAs on tomato fruits. BMC Genomics 13, 7. 22230737 10.1186/1471-2164-13-7 PMC3266637

