# Supplementary material for: RNA sequencing and functional analysis implicate the regulatory role of long non-coding RNAs in tomato fruit ripening
Source: J Exp Bot. 2015 May 6;66(15):4483–95. doi: 10.1093/jxb/erv203 (PMC4507755; doi:10.1093/jxb/erv203)
Supplement: Supplementary Data [file supp_66_15_4483__index.html]

RNA sequencing and functional analysis implicate the regulatory role of long non-coding RNAs in tomato fruit ripening — RNA sequencing and functional analysis implicate the regulatory role of long non-coding RNAs in tomato fruit ripening — Supplementary Data 

# RNA sequencing and functional analysis implicate the regulatory role of long non-coding RNAs in tomato fruit ripening

## Supplementary Data

Data files

**Files in this Data Supplement:**

- Supplementary Data - Supplementary Data
- Supplementary Data - Supplementary Data
- Supplementary Data - Supplementary Data
