# Supplementary material for: RNA sequencing and functional analysis implicate the regulatory role of long non-coding RNAs in tomato fruit ripening
Source: J Exp Bot. 2015 May 6;66(15):4483–95. doi: 10.1093/jxb/erv203 (PMC4507755; doi:10.1093/jxb/erv203)
Supplement: Supplementary Data [file supp_erv203_jexbot147322_file001.pdf]

Journal of Experimental Botany

RNA-seq and functional analysis implicate the regulatory role of long noncoding RNAs in tomato fruit ripening

Benzhong Zhu, YongFang Yang, Ran Li, Daqi Fu, Liwei Wen, Yunbo Luo* and Hongliang Zhu*
*Correspondence should be addressed. E-mail: hlzhu@cau.edu.cn and lyb@cau.edu.cn

Methods

Semi-quantitative reverse transcription PCR (Semi-quantitative PCR)

Reverse transcription (RT) was performed with TRV RT primer (Sha *et al.*, 2014). The level of *coat protein* (*CP*) gene was as internal control of PCR assay. PCR was performed using EasyTaq PCR SuperMix (Trans, Beijing, China) with PCR system T-100 (Bio-rad, CA, USA). PCR conditions for *CP* and *RIN*, lncRNA1459 or lncRNA1840 were as follows: 94 ℃ for 2 min, followed by 24 cycles of 94 ℃ for 30 s, 55 ℃ for 30 s and 72 ℃ for 30 s. All PCR data presented are representative of three independent experiments. Oligonucleotide primers used are listed in Supplementary Table S1 (avaliable at *JXB* online).

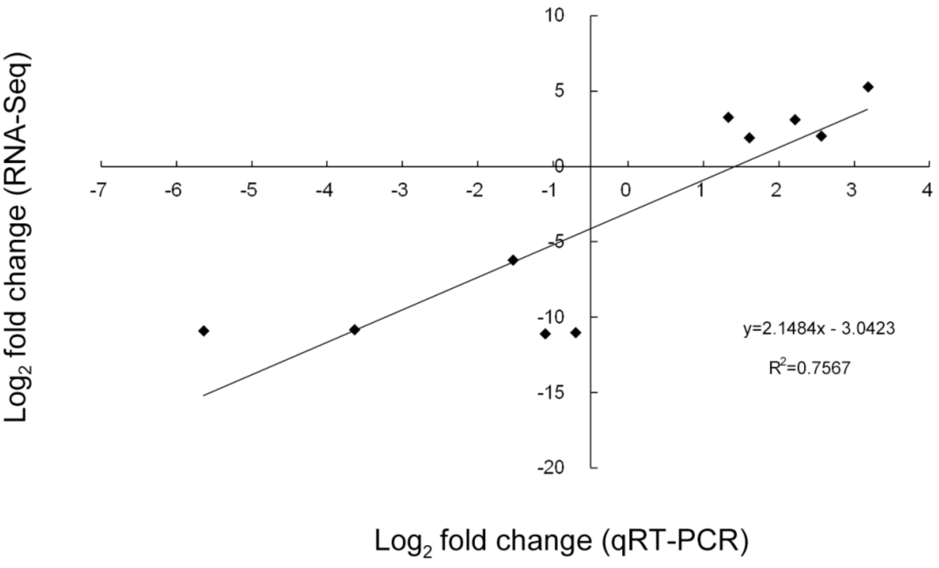

Fig. S1. Expression levels as determined by RNA-seq and qRT-PCR are highly correlated. The logarithm of fold change values in the RNA-seq and the qRT-PCR data were plotted along with the linear fit line to examine the correlation relationship between the two methods (R^2=0.76, $p<0.001$).

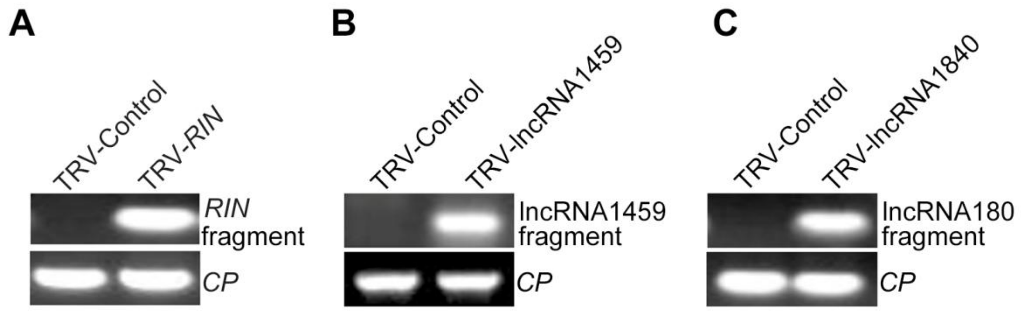

Fig. S2 Semi-PCR detection of recombinant TRV RNA in uninjected tomato fruits with infiltrated carpopodiums. RNA samples were extracted from TRV-*RIN*, TRV-lncRNA and TRV control tomato fruits, and PCR was performed with TRV2-*RIN*, TRV2-lncRNA and *CP* primers. (A) TRV-*RIN* . (B) TRV-lncRNA1459. (C) TRV-lncRNA1840.
